# Syngeneic Transplantation of Olfactory Ectomesenchymal Stem Cells Restores Learning and Memory Abilities in a Rat Model of Global Cerebral Ischemia

**DOI:** 10.1155/2018/2683969

**Published:** 2018-05-10

**Authors:** Antoine D. Veron, Cécile Bienboire-Frosini, Stéphane D. Girard, Kevin Sadelli, Jean-Claude Stamegna, Michel Khrestchatisky, Jennifer Alexis, Patrick Pageat, Pietro Asproni, Manuel Mengoli, François S. Roman

**Affiliations:** ^1^Aix Marseille Univ, CNRS, INP, Inst Neurophysiopathol, Marseille, France; ^2^IRSEA, Research Institute in Semiochemistry and Applied Ethology, 84400 Apt, France

## Abstract

Stem cells are considered as promising tools to repair diverse tissue injuries. Among the different stem cell types, the “olfactory ectomesenchymal stem cells” (OE-MSCs) located in the adult olfactory mucosa stand as one of the best candidates. Here, we evaluated if OE-MSC grafts could decrease memory impairments due to ischemic injury. OE-MSCs were collected from syngeneic F344 rats. After a two-step global cerebral ischemia, inducing hippocampal lesions, learning abilities were evaluated using an olfactory associative discrimination task. Cells were grafted into the hippocampus 5 weeks after injury and animal's learning abilities reassessed. Rats were then sacrificed and the brains collected for immunohistochemical analyses. We observed significant impairments in learning and memory abilities following ischemia. However, 4 weeks after OE-MSC grafts, animals displayed learning and memory performances similar to those of controls, while sham rats did not improve them. Immunohistochemical analyses revealed that grafts promoted neuroblast and glial cell proliferation, which could permit to restore cognitive functions. These results demonstrated, for the first time, that syngeneic transplantations of OE-MSCs in rats can restore cognitive abilities impaired after brain injuries and provide support for the development of clinical studies based on grafts of OE-MSCs in amnesic patients following brain injuries.

## 1. Introduction

Regenerating the central nervous system stands as a scientific challenge raising great hopes [1]. While some brain areas display lifelong neurogenesis [2], this process is unable to compensate the deleterious consequences of degenerative diseases or severe trauma. Therefore, the transplantation of exogenous stem cells has been proposed as an attractive approach to replace dead cells and/or to act as a neuroprotective agent [3–5]. However, the ethical and technical issues associated with embryonic, fetal, and neural stem cells limit the transfer of promising experimental results to clinical applications [6–8]. Accordingly, other stem cell types continue to be evaluated in preclinical studies to bypass these constraints.

Among the potential candidates for regenerative therapy, olfactory lamina propria stem cells are promising ones [9, 10]. Located in the lamina propria of the olfactory mucosa, they might partly support its permanently self-renewing capacity, although their physiological role is still not well established [11–13]. These cells, identified as a member of the mesenchymal stem cell family, display a high *in vitro* proliferation rate and were characterized as multipotent [9, 10, 14]. They also secrete neurotrophic and immunomodulatory factors which could protect injured brain areas [15, 16]. Due to their origin and characteristics, they are named “olfactory ectomesenchymal stem cells” (OE-MSCs) [9] and may provide a potential source of stem cells to regenerate injured central nervous system [12].

Interestingly, these cells can be easily collected in humans [17]. Indeed, the olfactory lamina propria can be harvested in every individual under local anesthesia, which could allow autologous transplantation. Moreover, OE-MSCs have been successfully evaluated in different rodent models, including myocardial infarct [18], spinal cord trauma [19–21], cochlear damage [22], Parkinson's disease [23], and chemically induced hippocampus injuries [24]. It should be noted that most of these studies relied on the use of xenotransplantation of human OE-MSCs in rodent models, limiting the evaluation of the full therapeutic potential. Altogether, these singular properties could overcome all the concerns that are usually encountered with most other stem cell types and promote the usefulness of OE-MSC transplantation.

In the present study, we evaluated for the first time, using a syngeneic approach, the therapeutic potential of delayed OE-MSC grafts in a rat model of global cerebral ischemia (GCI). This model mimics the effects of cardiac arrest/asphyxia in human and particularly the neurologic damage within the hippocampus [25–28]. Located in the medial temporal lobe, the hippocampus is a vulnerable structure which plays a central role in cognitive processes. Thus, the loss of hippocampal neurons, consecutive to trauma, age-related diseases, or cardiac arrests, induces severe learning and memory deficits [29–31]. Here, we show that delayed OE-MSC grafts after GCI-induced hippocampal injury restored learning and memory abilities and promote neurogenesis.

## 2. Material and Methods

### 2.1. Experimental Design

Ischemic episode is defined as day 1. Hippocampal lesions were behaviorally controlled using an olfactory associative discrimination task at week 4. Syngeneic transplantations of OE-MSCs were performed at week 5. Learning and memory abilities of sham and grafted animals were then retested at week 9, before sacrificing the animals for immunohistological analyses. Anesthesia and surgical procedures were conducted in accordance with the law of animal experimentation as outlined in the European Community Council Directive (2010/63/UE); the protocols were approved by the Animal Care Committee of Aix-Marseille University. All efforts were made to minimize animal suffering and to reduce the number of animals used while complying with statistical constraints.

### 2.2. Animals

The study was performed with syngeneic male individuals (Fischer 344, Charles River Laboratories, France, *n* = 30). Rats were housed in individual cages and supplied with food and water ad libitum, except when tested using an olfactory associative discrimination task. The housing temperature was constant (22°C) under a 12 h/12 h light-dark cycle (lights on at 6:30 am).

### 2.3. Collection and Expansion of Rat OE-MSCs

Nasal olfactory mucosae were obtained from six 3-week-old rats using the procedures previously described [17, 32]. Rats were deeply anesthetized with an overdose of sodium pentobarbital (Nembutal, Centravet, France, 120 mg/kg, ip) and decapitated. After isolation of the head, skin, facial muscles, and bone covering, the nasal cavity was removed. The olfactory mucosa was then collected from both sides using a sterile needle, and fragments were pooled into a sterile 2 mL tube filled with 37°C DMEM/Ham's F12 culture medium supplemented with 10% fetal bovine serum, 200 units/mL of penicillin, 200 *μ*g/mL of streptomycin (all products from Life Technologies, France), 1.25 *μ*g/mL amphotericin B (Fungizone, Sigma-Aldrich, France), and 12.5 *μ*g/mL of Plasmocin treatment (InvivoGen, France). After the biopsy, pieces of olfactory mucosa were washed twice in the same medium, dissected into small parts (~2 mm^2^), and plated on poly-L-lysine-coated plastic dishes (5 *μ*g/cm^2^, Sigma-Aldrich) in 250 *μ*L of the same medium. Two weeks later, concentrations of antibiotics were reduced (100 units/mL of penicillin and 100 *μ*g/mL of streptomycin), amphotericin B was removed, and Plasmocin treatment was replaced by 1.25 *μ*g/mL Plasmocin prophylactic (InvivoGen). This medium is referred to as growth medium throughout the manuscript and was gently renewed every 2 to 3 days. When confluency was reached, the cells were detached using a trypsin-EDTA solution (0.05%, Life Technologies), pooled and centrifuged at 300 ×g, for 5 min and replated without exceeding a 1 : 3 cell split ratio.

### 2.4. Flow Cytometry Analysis

Flow cytometry analysis was carried out as previously described [32]. Cells were washed twice with phosphate-buffered saline (PBS) and then harvested using TrypLE™ Select Enzyme (Life Technologies). The cells were centrifuged (300 ×g, 5 min), resuspended in cold blocking solution (10% fetal bovine serum in PBS), and centrifuged again. Cells were paraformaldehyde-fixed for 15 min RT (4%, Antigenfix, Microm Microtech, France), washed twice in blocking solution, and permeabilized in cold methanol (90%, −20°C) for 30 min at 4°C, before being washed twice again. After this, cells were incubated 20 min RT with primary antibodies against CD34, CD44, or CD73 (Table 1) diluted in blocking solution or incubated, as a negative control, with the corresponding isotype control (rabbit IgG, Abcam, France) at the same concentration. Cells were then washed three times by centrifugation (600 ×g, 5 min) and incubated with the corresponding secondary antibody (Jackson ImmunoResearch, United Kingdom, 1 : 500, see Table 1) diluted in blocking solution for 20 min RT in the absence of light. After three washes, cells were immediately processed for flow cytometric analysis. Acquisitions were performed on flow cytometer (a FACSCanto II, BD Biosciences, France) using BD FACSDiva software. At least 10,000 events were recorded for each analysis, and measures were performed in duplicate. Percentages are presented after the subtraction of isotype background and refer to the total living population analyzed.

### 2.5. Immunocytochemistry

OE-MSCs were plated on glass coverslips at a density of 15,000 cells per cm^2^ in growth medium for 48 h. Cells were then paraformaldehyde-fixed for 15 min and incubated for 1 hour at RT with blocking buffer (3% bovine serum albumin, 5% goat serum, and 0.1% Triton X-100, all from Sigma-Aldrich) in PBS. Glass coverslips were then incubated for 90 min at RT with the appropriate primary antibody diluted in blocking solution (Table 1). After 3 washes, cells were incubated for 60 min with the appropriate secondary antibody (see Table 1) and washed 3 times. Cells were finally counterstained with 0.5 *μ*g/mL Hoechst blue (33258, Sigma-Aldrich) for 10 min and coverslips mounted with antifading medium (ProLong Diamond, Life Technologies). Control conditions were carried out by omitting the primary antibody.

### 2.6. Generation of OE-MSCs Expressing GFP

To generate cells stably expressing the green fluorescent protein (GFP), 40 million cells were electroporated (Neon® Transfection System, Life Technologies) with HSC1-GiP EiP-GFP plasmid [33], which allows expression of GFP along with a puromycin resistance gene, under the control of EF1*α* promoter. Optimization protocol 2 was used (1400 volts, 20 ms, 1 pulse), and then cells were seeded in a 25 cm^2^ flask in growth medium without any antibiotics for 24 h. On the next day, antibiotics were added to the medium, and 48 h after electroporation, cells were harvested and reseeded in the same flask in growth medium with 4 *μ*g/mL puromycin for two days and at 2 *μ*g/mL until all non-GFP cells were dead.

### 2.7. Ischemic Model

Transient global cerebral ischemia was induced in 10-week-old male F344 rats (*n* = 16) weighing 250 grams, using the four-vessel occlusion method (4VO) described by Pulsinelli and Brierley [25, 34]. This model was adapted for a two-step process to better control variability of ischemia, and detailed methods were already reported [28]. Due to vulnerability of the Fischer 344 strain, a 7-day interval was managed between both steps [35, 36]; both vertebral arteries were thermos-cauterized, and 1 week later, an ischemic episode was induced by clamping carotid arteries on experimental day 1.

### 2.8. Behavioral Procedure

Three weeks after cerebral ischemia and four weeks after OE-MSC transplantation, the rats' learning and memory abilities were assessed using the olfactory associative task [37, 38]. Rats underwent progressive water restriction one week before the test. They were then trained to make two scent-reward associations in a rectangular box. One session consisted of 40 trials using a successive Go/No-Go paradigm. Individual trials were run in a quasi-random fashion (no more than 3 consecutive trials with the same valence). When the positive scent (S+) was delivered into the cage, responding rats were rewarded with 0.1 mL of water by going to the water port. If the rats repeated this behavior in response to the delivery of the negative scent (S−), they did not receive water but an unpleasant bright light in the corner of the cage. A mean of 80 ± 5% correct responses was required to ensure that all animals had learned both associations. Whether or not the animals responded to the scent presentation (a trial), a fixed intertrial interval (ITI) of 15 seconds with clean air was initiated. If a response was given during the last second of the ITI, the next trial was delayed by 10 seconds and so on. The mean ITI was the number of seconds added to the fixed 15 s, divided by the number of ITIs, which in this experiment amounted to 39.

### 2.9. Transplantation Surgery

Four weeks after ischemia, rats with severe learning and memory deficits were randomly assigned to 2 groups. In the transplanted group (grafted, *n* = 6), rats received bilateral grafts of olfactory stem cells (1,000,000 cells in total), while in the nontransplanted group (sham, *n* = 6), animals received an equal amount of culture medium without cells. Anesthetized rats were inserted in a stereotaxic frame, the skull surface was exposed, and holes were drilled at the appropriate site. Cell suspensions or vehicle was injected with a 1 *μ*L Hamilton syringe connected to a stereotactic syringe pump (KDS 310, KD scientific, USA) into both hippocampi. Anteroposterior (AP), lateral (L), and vertical (V) coordinates for microinjection were taken relative to the bregma: (injection 1: AP—3.1; L ± 3; V—2.8), (injection 2: AP—3; L ± 2.4; V—3), and (injection 3: AP—3.8; L ± 2.6; V—3). The infused volume was 1 *μ*L per injection site, and the rate of infusion was 0.5 *μ*L/min. Rats were then allowed to recover 4 weeks until behavioral analysis.

### 2.10. Immunostaining

Immunohistochemistry was carried out to identify the presence of injected cells after transplantation and to follow their differentiation. After the last behavioral session, rats were deeply anesthetized with an injection of sodium pentobarbital (120 mg/kg, ip) and intracardially perfused with PBS, followed by 4% paraformaldehyde. The brains were then frozen, and coronal sections (40 *μ*m thick) were acquired. Three sections of each brain (forward, over, and backward of the injection site) were used for each antibody (Table 1). The rest of the procedure was the same as for immunocytochemistry described above.

### 2.11. Quantification

An inverted microscope (Axio Imager, Carl Zeiss microscopy, Germany) was used to acquire 10x magnification pictures that were then treated with ImageJ. Pictures were first converted into binary images; appropriate thresholds were determined for each antibody, and percentages of reactive pixels were quantified within selected hippocampal area. Thus, six values were acquired per rat for each antibody, as suggested by previous studies [39, 40].

### 2.12. Data Analysis and Statistics

All data are presented as means ± SEM. Statistical analyses were performed with SPSS/PC+ statistics 11.0 software (SPSS Inc., USA). Behavioral responses were analyzed using a repeated measures MANOVA. Then, subsequent ANOVAs for each session were computed. The comparison between grafted and sham groups according to histological parameters was carried out using Student's *t*-test or Wilcoxon two-sample test depending on normality and variances (homogeneity of variances was verified using Fisher's test).

## 3. Results

### 3.1. Characterization of Rat OE-MSCs

**Figure 1:**
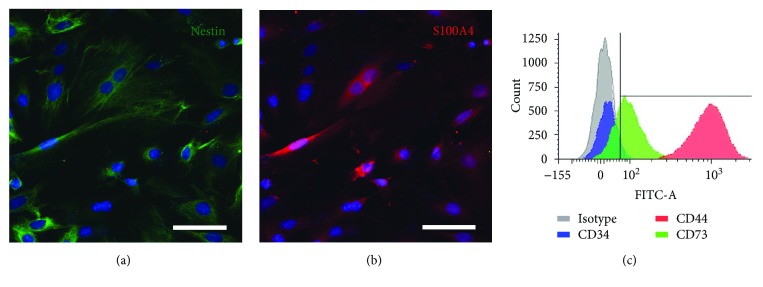
Characterization of rat OE-MSCs *in vitro*. Prior to engraftment, immunochemistry revealed that 100% of cells expressed the nestin (a, green) and S100A4 (b, red) proteins. OE-MSCs were immunostained with 3 surface markers, quantified using a flow cytometer and expression level compared to isotype (c). Nuclei were counterstained with Hoechst. Scale bar: 200 *μ*m (a–c).

The entire population of our cells expressed the nestin (Figure 1(a)) and S100A4 (Figure 1(b)) proteins. Using flow cytometry, we analyzed expression of 3 surface markers. CD34 expression was extremely low (4.1%) while CD44 was highly and homogeneously expressed (98.5%), and CD73 expression was moderate (62.6%) (Figure 1(c)). All OE-MSCs stably expressed GFP just before graft, and electroporation technique did not modify expression of nestin and S100A4 proteins (data not shown).

### 3.2. Behavioral Studies

#### 3.2.1. Preengraftment

**Figure 2:**
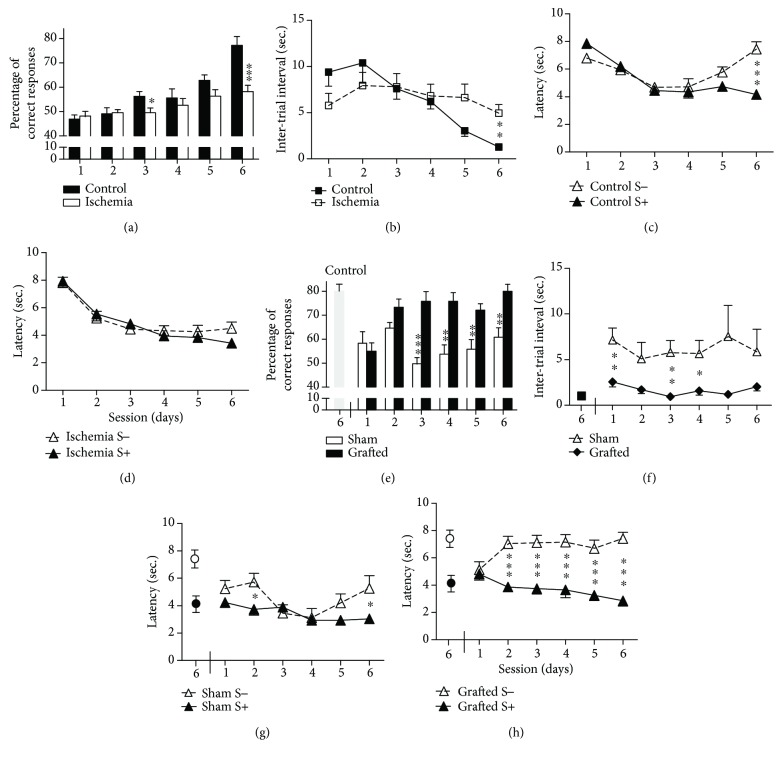
Assessment of hippocampus-dependent learning and memory abilities after 15 minutes of global cerebral ischemia and after OE-MSC transplantation. Cognitive abilities of rats were assessed in the olfactory associative task 4 weeks after surgery. (a) Mean percentage of correct responses, (b) intertrial interval, and (c and d) latencies were obtained during 6 training sessions of 40 trials per day. Ischemic rats (*n* = 12) exhibited significant impairment in an associative memory task when compared with control rats (*n* = 8). The ischemia group was randomly divided between grafted (*n* = 6) and sham (*n* = 6) groups, and learning and memory abilities of rats were assessed 5 weeks after transplantation of OE-MSCs or culture medium. Performances obtained by control rats (*n* = 8) in session 6 served as the reference for the effectiveness of the memorization process. (e) Contrary to sham, grafted rats reached a percentage of correct answers comparable to control rats from day 2 and kept their high score until the end of the training sessions. The same improvement was observed for the intertrial interval (f) and latencies (g and h) only in grafted rats. ^∗^*p* < 0.05; ^∗∗^*p* < 0.01; ^∗∗∗^*p* < 0.001. S−: negative scent; S+: positive scent.

Prior to engraftment, behavioral performance was evaluated between control (*n* = 8) and ischemic (*n* = 12) rats. Analyses of the percentage of correct responses (Figure 2(a)) showed that only control rats improved their performance across the six sessions [*F*(5,90) = 24.76; *p* < 0.001] with a substantial difference between the two groups in session 6 [*F*(1,18) = 19.09; *p* < 0.001].

The intertrial interval (Figure 2(b)) decreased across sessions in the control group, but not for the ischemia group, which performed at a constant level throughout all sessions. Consequently, a significant difference was observed across the six sessions [*F*(5,90) = 9.25; *p* < 0.001], with a significant group difference at session 6 [*F*(1,18) = 10.26; *p* < 0.05].

Performance analyzed in terms of S+ and S− latencies (Figures 2(c) and 2(d)) showed a significant impairment in the ischemia group. In the control group, correct discriminative associations were observed in the sixth session [*F*(1,18) = 27.12; *p* < 0.001]. The ischemia group showed a gradual decrease in latencies across the six sessions, but for both stimuli. Consequently, a significant difference between groups was observed on S− across the six sessions [*F*(5,90) = 13.67; *p* < 0.001], but not on S+ stimuli.

#### 3.2.2. Postengraftment

The ischemia group was randomly divided between grafted and sham groups. Performances obtained by control rats in session 6 served as the reference for the effectiveness of the memorization process.

The performance of grafted rats improved across sessions, reaching a correct response score of 80 ± 5% in session 3 (Figure 2(e)). Analyses indicated differences across sessions [*F*(5,50) = 7.12; *p* < 0.001], and grafted rats displayed better performance than sham rats since session 3 [*F*(1,10) ≥ 11.1; *p* ≤ 0.01]. Performances of grafted rats differed from that of the controls (assessed in the previous session 6) only during the first session [*F*(1,12) = 16.92; *p* < 0.001], while this difference was consistently observed for the sham rats, including the last session [*F*(1,12) ≥ 7.38; *p* ≤ 0.05].

The intertrial interval decreased for the grafted rats when compared to the first session (Figure 2(f)). The sham group still displayed higher intertrial interval times, and significant differences were present between the grafted and sham groups during sessions 1, 3, and 4 [*F*(1,10) ≥ 7.08; *p* ≤ 0.05].

Training performance analyzed by S+ and S− latencies for the two groups is presented in Figures 2(g) and 2(h). Sham rats responded to S+ or S− trials without significant discrimination, except on sessions 2 and 6 (*p* < 0.05) (Figure 2(g)). While, correct associations of S+ and S− stimuli started to be significant from the second session for the grafted group [*F*(1,10) ≥ 20.01; *p* ≤ 0.001] (Figure 2(h)). A difference between the two groups in response to S− was observed over the sessions [*F*(5,50) = 4.91; *p* < 0.001], but such difference was not seen for the S+ stimuli.

### 3.3. Rat Syngeneic OE-MSCs Promote Neuroblast Proliferation in Hippocampi

**Figure 3:**
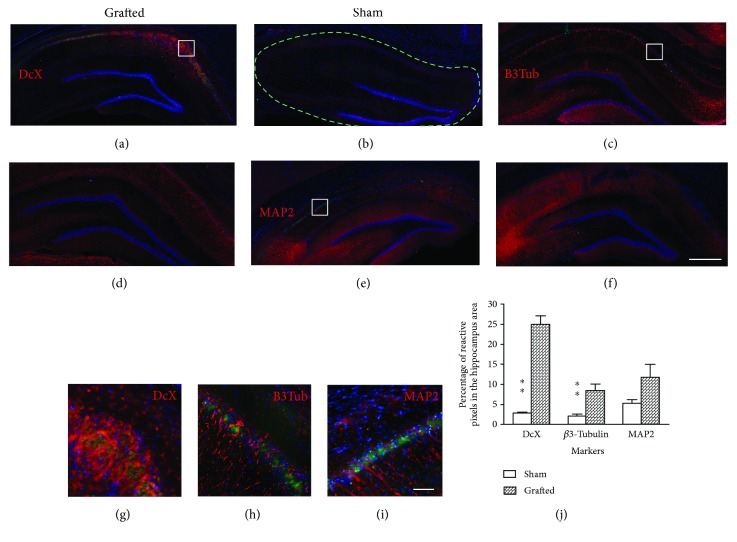
Assessment of hippocampal neurogenesis after OE-MSC transplantation in ischemic rats. Immunohistological analyses revealed an increase of neurons in the hippocampus of grafted animals. (a) Newborn neurons expressing DcX (red) were mostly observed in the CA1 and CA2 areas of grafted animals, but none was observed in sham animals (b). Mature neurons expressing (red) *β*3-tubulin (c and d) or MAP2 (e and f) were also present in the hippocampi of animals from both groups. Higher magnification images revealed that no cells express both GFP (green) and tested neural markers (red) (g–i). The number of DcX and *β*3-tubulin-positive cells was significantly higher in the grafted group when compared to sham, and a tendency is observed for the MAP2 marker (*p* = 0.69) (j). Each image is representative of different animals from both groups. Scale bar: 1 mm (a–f), 100 *μ*m (g–i). ^∗∗^*p* < 0.01. DcX: doublecortin; B3Tub: *β*3-tubulin; MAP2: microtubule-associated protein 2. Dashed line: selected area for antibody quantification.

Five weeks after graft, only few GFP cells were observed and they displayed an apoptotic morphology. However, there was a significant increase of cells expressing doublecortin (DcX), a protein expressed by neuronal precursor cells and immature neurons, in hippocampi of the grafted group when compared to the sham group (*p* < 0.01) (Figures 3(a), 3(b), and 3(j)). Interestingly, the same difference was observed for the *β*3-tubulin marker (*p* < 0.01) (Figures 3(c), 3(d), and 3(j)), a protein involved in neural development and expressed by maturing neurons. There was no significant difference regarding the MAP2 marker, a protein involved in microtubule assembly, but a tendency was observed (*p* = 0.06) (Figures 3(e), 3(f), and 3(j)). No colocalization was observed between GFP cells and neuronal markers (Figures 3(g)–3(i)).

### 3.4. Syngeneic Transplantation of Rat OE-MSCs Stimulates Glial Reaction in Hippocampus of Ischemic Rats

**Figure 4:**
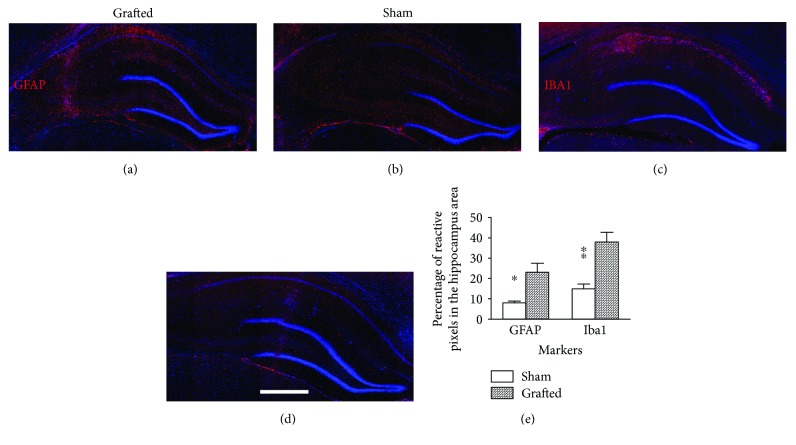
Assessment of glial reaction in the hippocampus after OE-MSC transplantation in ischemic rats. Immunohistological analyses revealed an increase of glial cells in the hippocampus of grafted animals. We found a higher number of GFAP (a and b) and Iba1 (c and d) reactive cells in hippocampi of grafted animals in comparison to sham rats (e). Each image is a representative of different animals from both groups. Scale bar: 1 mm. ^∗^*p* < 0.05; ^∗∗^*p* < 0.01. GFAP: glial fibrillary acidic protein; Iba1: ionized calcium-binding adaptor molecule 1.

Five weeks after the injection of OE-MSCs, we observed an accumulation of glial cells in the hippocampus of grafted animals. Astrocytes were spread in all parts of the hippocampus (Figure 4(a)). Conversely, the accumulation of microglia was restricted to the neuronal layers of CA1 and CA2 (Figure 4(c)). This increase of glial cells was not observed in sham animals (Figures 4(b) and 4(d)), and quantification revealed a significantly higher amount of GFAP (*p* < 0.05) and Iba1 (*p* < 0.01) reactive cells (Figure 4(e)) in grafted rats.

## 4. Discussion

The present study demonstrates that engraftment of syngeneic OE-MSCs following GCI restored impaired learning and memory abilities. The results also showed a significant increase of newborn neurons in the hippocampus of grafted animals, like demonstrated by other studies leading stem cell grafts after stroke [41, 42]. As not that much newborn neurons were observed in sham animals, we can assume that this neurogenesis is related to the transplantations of OE-MSCs. Unfortunately, our data does not allow us to definitively determine whether these newborn neurons originate from the differentiation of injected OE-MSCs or from endogenous neurogenesis.

This neurogenic effect was associated with a remarkable recovery of cognitive function. Global cerebral ischemia leads to specific neuron losses in the hippocampus and induces learning and memory deficits, especially in this olfactory associative discrimination task [28]. Ischemic animals reached only 60% of correct answers, against 80% for control, and were unable to make the cue-reward association. Impressively, 4 weeks after the transplantation, these same rats but grafted with OE-MSC were able to learn these associations, demonstrating latencies and percentages of correct answers similar to those obtained by naive control animals. Meanwhile, the performance of sham animals did not improve over time, and their results on the first and second set of sessions were nearly identical. This suggests that the restoration of learning and memory abilities was mainly related to OE-MSC transplantation.

Though we did not assess the functionality of newborn neurons, the recovery of learning abilities could be linked to the number of immature DcX-positive neurons in the hippocampus. Previous studies reported that immature neurons could achieve a more robust long-term potentiation than mature dentate granule cells [43, 44], which is crucial for memorizing information and learning [45–47]. In our study, we observed a twelvefold increase in DcX-positive cells, mainly around the CA1 area of the hippocampi, which may have contributed to restore plasticity and related learning and memory processes.

Surprisingly, we observed a long-lasting glial reaction, five weeks after the transplantation of OE-MSCs. While mesenchymal stem cells display hypoimmunogenicity and immunosuppressive activities [15, 48, 49], it is indisputable that the syngeneic OE-MSC engraftment led to microglial recruitment. It could be of interest to evaluate if the same pattern would be observed in case of autologous grafts. However, recent findings have also demonstrated that microglia may have a positive effect on neurogenesis and neuroprotection [39, 40, 50, 51]. In addition, preliminary results also show nestin-positive cells displaying glial morphology. This protein is expressed by radial glia [52], which are implicated in neurogenesis and migration [53]. Moreover, characterization of cells revealed that expressions of surface markers CD34, CD44, and CD73, and proteins nestin and S100A4 were similar to data previously obtained in OE-MSCs from humans, rats, and other mammalian genera [9, 32]. Other studies using central nervous system injury models also suggest that paracrine signaling may have mediated the therapeutic effect after human OE-MSC transplantation [19–22, 24]. Thus, we could hypothesize that OE-MSCs may have stimulated endogenous neurogenesis by secreting various trophic factors [49, 54], but also by interacting with microglia and induce them in a neuroprotective phenotype, like already proposed as features of MSCs [55].

## 5. Conclusion

This study demonstrated, for the first time, that OE-MSC grafts in syngeneic rats allowed learning and memory recoveries after GCI. These cells avoid the main technical and ethical issues associated with other stem cell types. They can be easily collected, including in human, and displayed a high proliferation rate, allowing them to be transplanted quickly following injury. Together, these results pave the way for the development of clinical studies based on autologous grafts of OE-MSCs in patients with posttraumatic memory loss.

## Figures and Tables

**Table 1:** Antibodies used for immunochemistry and flow cytometry.

Target	Host	Supplier	Dilution	Secondary antibody
GFP	Rabbit polyclonal	Millipore	1 : 250	488 anti-rabbit
NeuN	Mouse monoclonal	Millipore	1 : 250	594 anti-mouse
GFAP	Rabbit polyclonal	Dako	1 : 500	594 anti-rabbit
Iba1	Rabbit polyclonal	Wako	1 : 500	594 anti-rabbit
DcX	Rabbit polyclonal	Abcam	1 : 300	594 anti-rabbit
*β*3-Tubulin	Mouse monoclonal	Sigma	1 : 250	594 anti-mouse
MAP2	Chicken monoclonal	Abcam	1 : 250	594 anti-chicken
CD34	Rabbit polyclonal	Abcam	1 : 50	488 anti-rabbit
CD44	Rabbit polyclonal	Abcam	1 : 50	488 anti-rabbit
CD73	Rabbit polyclonal	Abcam	1 : 110	488 anti-rabbit

GFP: green fluorescent protein; NeuN: neuronal nuclei; GFAP: glial fibrillary acidic protein; Iba1: ionized calcium-binding adaptor molecule 1; DcX: doublecortin; MAP2: microtubule-associated protein 2.

## Abbreviations

DcX: Doublecortin
GCI: Global cerebral ischemia
GFAP: Glial fibrillary acidic protein
GFP: Green fluorescent protein
MAP2: Microtubule-associated protein 2
MSC: Mesenchymal stem cell
OE-MSC: Olfactory ectomesenchymal stem cell
PBS: Phosphate-buffered saline.
